# Genetic Variation in Genes Encoding Airway Epithelial Potassium Channels Is Associated with Chronic Rhinosinusitis in a Pediatric Population

**DOI:** 10.1371/journal.pone.0089329

**Published:** 2014-03-03

**Authors:** Michael T. Purkey, Jin Li, Frank Mentch, Struan F. A. Grant, Martin Desrosiers, Hakon Hakonarson, Elina Toskala

**Affiliations:** 1 Department of Otorhinolaryngology, Hospital of the University of Pennsylvania, Philadelphia, Pennsylvania, United States of America; 2 Center for Applied Genomics, the Children's Hospital of Philadelphia, Philadelphia, Pennsylvania, United States of America; 3 Division of Human Genetics, the Children's Hospital of Philadelphia, Philadelphia, Pennsylvania, United States of America; 4 Department of Otolaryngology, Montreal General Hospital, McGill University, Montreal, Québec Canada; 5 Department of Pediatrics, Perelman School of Medicine, University of Pennsylvania, Philadelphia, Pennsylvania, United States of America; 6 Department of Otolaryngology, Temple University, Philadelphia, Pennsylvania, United States of America; Kunming Institute of Zoology, Chinese Academy of Sciences, China

## Abstract

**Background:**

Apical potassium channels regulate ion transport in airway epithelial cells and influence air surface liquid (ASL) hydration and mucociliary clearance (MCC). We sought to identify whether genetic variation within genes encoding airway potassium channels is associated with chronic rhinosinusitis (CRS).

**Methods:**

Single nucleotide polymorphism (SNP) genotypes for selected potassium channels were derived from data generated on the Illumnia HumanHap550 BeadChip or Illumina Human610-Quad BeadChip for 828 unrelated individuals diagnosed with CRS and 5,083 unrelated healthy controls from the Children's Hospital of Philadelphia (CHOP). Statistical analysis was performed with set-based tests using PLINK, and corrected for multiple testing.

**Results:**

Set-based case control analysis revealed the gene *KCNMA1* was associated with CRS in our Caucasian subset of the cohort (598 CRS cases and 3,489 controls; p = 0.022, based on 10,000 permutations). In addition there was borderline evidence that the gene *KCNQ5* (p = 0.0704) was associated with the trait in our African American subset of the cohort (230 CRS cases and 1,594 controls). In addition to the top significant SNPs rs2917454 and rs6907229, imputation analysis uncovered additional genetic variants in *KCNMA1* and in *KCNQ5* that were associated with CRS.

**Conclusions:**

We have implicated two airway epithelial potassium channels as novel susceptibility loci in contributing to the pathogenesis of CRS.

## Introduction

Chronic rhinosinusits (CRS) is a spectrum of clinical disease characterized by persistent inflammation of the nasal cavity and paranasal sinuses [1]. Historically, CRS was considered an extension of acute bacterial sinusitis and the role of bacterial infection was considered primary in the development of the disease. Recent research and clinical focus has shifted from a primary infectious etiology (in most cases) to chronic mucosal inflammation as the primary underlying dysfunction in CRS [2]. Despite advances in our understanding of the cellular and molecular changes in the nasal mucosa of patients with CRS, the underlying pathophysiology remains incompletely understood and likely includes many different pathophysiologic mechanisms that result from a complex underlying genetic predisposition that is subsequently influenced by environmental effects.

Multiple clinical observations of the cause of chronic mucosal inflammation drive the rationale for current medical and surgical treatment of the disease and include anatomic osteomeatal complex obstruction, allergy associated inflammation, osteitis, MCC dysfunction, and advanced microbial defense mechanisms such as biofilm formation [3]. Despite our attempts to classify patients based on clinical and radiographic findings, most cases of CRS are currently considered idiopathic and our treatment algorithms are variable in their efficacy for a given patient [4].

Classic studies of heritability for CRS have not been performed, however a genetic basis of disease has been suspected for many reasons [5]. Familial aggregation of disease points to an underlying genetic basis and many reports have shown unusually high prevalence of CRS within families [6]–[8]. Patients with CRS are also more likely to report a positive family history [9]. Observations of the sinonasal manifestations of primary ciliary dyskinesia and cystic fibrosis (CF) raise the possibility that the development of CRS may have a common genetic etiology. Others have shown an increased prevalence of CRS in carriers of the cystic fibrosis transmembrane conductance regulator (CFTR) mutation [10], [11]. Further, there is evidence that the inflammatory profiles in CRS are similar to allergic rhinitis and asthma, two complex diseases with a well-established genetic basis [12].

Cystic fibrosis is the classic example of a monogenic disease producing a CRS phenotype. In the case of CF, the almost universal and often severe sinus disease that develops has been long recognized [13]. In CF patients, changes in the CFTR gene on chromosome 7 result in dysfunctional ion transport of chloride across the apical membrane of exocrine glandular cells, including airway epithelium, and subsequent ASL dehydration develops [13], [14]. This environment is susceptible to chronic bacterial colonization and infection which triggers a host response and leads to a severe phenotype of CRS. The presence of a single gene defect causing ion dysregulation that leads to CRS is a striking observation. We are interested in elucidating whether this observation has implications for the early molecular changes in the non-CF adult patient who develops CRS.

Recent work has been performed examining the role of various potassium channels and their relationship to ion transport in bronchial epithelial cells. Potassium channels are known to be functional at both the basolateral and apical membrane of airway epithelium [15]. The collective function of these channels is to mediate potassium currents of diverse electrophysiological and regulatory properties. The role of basolateral potassium channels to regulate membrane potential and maintain an electrochemical driving force for transepithelial ion and liquid transport is well established [16]. Understanding the role of apical potassium channels and exploring the possibility of utilizing them as a therapeutic target for diseases characterized by abnormal ion transport is an emerging field [17]. An exciting clinical example of the potential of ion transport modulation is the recent success of the CFTR potentiating agent, ivacaftor, in the treatment of CF. This investigational, orally administered drug augments CFTR function and has been shown to produce sustained improvement in multiple clinical outcome measures in patients with CF while possessing an acceptable safety profile [18]–[20].

Three main classes of apical epithelial potassium channels have been identified in respiratory epithelium. These include large conductance Ca^2+^-activated and voltage-dependent 6-TMD (KCa and Kv), 2-pore 4-TMD (K2P), and inward rectified 2-TMD potassium channels (Kir) [21]. Evidence suggests that all three classes may have the ability to modulate ion transport at the epithelial border, and subsequently impact air surface hydration, and therefore MCC. Large conductance, Ca^2+^-activated, and voltage-dependent BK channels have specifically been shown to contribute to an apical loop current favoring apical chloride efflux and maintenance of ASL hydration [22]. Members of the Kir family have been shown to impact trans-epithelial transport of sodium and chloride ions in addition to modulating amiloride-sensitive sodium channel (ENaC) and CFTR expression [23]. Other studies have shown that K2P channels are expressed and functionally active at the apical membrane of the airway epithelium [17].

The aim of this study is to identify whether genetic variation within genes encoding known airway potassium channel genes is associated with chronic sinusitis in a pediatric population.

## Methods

### Study subjects

In our study, 828 unrelated individuals diagnosed with CRS and 5,083 unrelated controls without CRS were included. Following genotyping, principal component analysis was conducted using EIGENSTRAT [24], in which 230 CRS cases and 1,594 controls were identified as African Americans; 598 CRS cases and 3,489 controls were identified as Caucasians. Further analyses were performed in these two ethnicity groups separately.

Patients included in this study had a history of CRS diagnosed by a Pediatric Otolaryngologist at CHOP using previously published criteria [25] that included history and physical exam findings consistent with CRS. Common symptoms of CRS included rhinorrhea, nasal congestion, facial pain, and post nasal drip. The duration of symptoms was greater than 12 weeks in all patients. Anterior rhinoscopy or flexible nasal endoscopy was performed in all patients. While not required for the diagnosis of CRS in children, CT imaging of the sinuses was used as an ancillary test in 46.2% of patients to confirm the diagnosis of CRS. All patients underwent allergy and immunological testing when clinically indicated as well as exclusion of CF when appropriate. Further phenotype refinement was performed to exclude CF patients as described below.

All recruited individuals filled in detailed questionnaires of medical history, medication and demographic data. Data were stored in a fully-integrated electronic medical record of the study subjects. Parental DNA was available for approximately 20% of the study participants. The institutional review board (IRB) of the Children's Hospital of Philadelphia specifically approved this study. Adults (>18 years) included in this study provided informed written consent using consent guidelines/procedures approved by the IRB of the Children's Hospital of Philadelphia. Written consent for children/minors included in the study was obtained from appropriate legal guardians with strict adherence to consent guidelines/procedures approved by the IRB of the Children's Hospital of Philadelphia.

### SNP genotyping

DNA extraction from whole blood and SNP genotyping was performed at the Center for Applied Genomics, CHOP. CRS cases and control samples were genotyped on Illumnia HumanHap550 BeadChip or Illumina Human610-Quad BeadChip, which share 535,752 common SNP markers. Because we are interested in airway potassium channel genes, we focused on the surprisingly large collection of potassium channel genes that have been identified in the literature [21]. Since important transcriptional or translational regulators could reside upstream or downstream of a gene, we extracted genotyping information on SNPs which are located within 20 kb upstream and downstream of the tested potassium channel genes.

### CFTR analysis

It is well known that CFTR has many subtle variants that contribute to pediatric rhinosinusitis. As such, CF is a significant confounding factor that needs to be cautiously identified and removed in a non CF CRS genetics study. We sought to identify all CF patients in our study population and exclude them from subsequent analysis. CF cases within our initial study population were identified by searching the EMR for the ICD code 277. Newborn screening for CF is routine in Pennsylvania and surrounding states. This test is highly sensitive. When combined with the high specificity of sweat chloride testing we are able to identify the overwhelming majority of CF cases in our population. We initially identified an additional 47 CF CRS cases that met study criteria and performed a genome-wide association study (GWAS) that included both CF and non CF CRS populations as described above. Allelic tests implemented in PLINK [26] were used to examine the genotype-phenotype association. In the Caucasian cohort, SNPs located close to or within the CFTR gene were highly significant in this GWAS analysis. However, such association was remarkably ablated when the CF CRS patients were removed (Table S1). This supports that CF is a significant confounding factor that needs to be cautiously identified and removed in a non CF CRS genetics study. The loss of CFTR association with CRS is strong evidence for accurate phenotype identification of the subsequent non CF CRS population that underwent further analysis. Of note, SNPs in the CFTR gene did not show significant association with the CRS phenotype in our African American cohort, with only one SNP with a p-value<0.05 (rs11978434, p-value = 0.0294).

### Statistical analysis

We excluded SNP markers which had a call rate <95%, minor allele frequency <0.01 or significant deviation from Hardy-Weinberg equilibrium (*P*<0.0001). Allelic tests were performed for each SNP using PLINK [26]. Set-based tests were carried out for 44 genes using PLINK. The two most significant independent SNPs (r^2^ threshold = 0.1) with p-value<0.01 were selected for each set. The mean of the selected SNP statistics was used to represent the statistics of the gene set. Permutations of case/control status were performed 10,000 times to obtain the null distribution of the statistics for each gene set under the hypothesis of no genetic association. Empirical p-values for each gene set were generated by comparing the observed test statistics and the ones based on permutations [26]–[28]. Multiple-testing corrected p-values were generated based on Bonferroni correction for 44 tests. In our imputation analysis via software package IMPUTE2 [29], [30], we used the 1000 Genome Phase I integrated variant set as reference panel (http://mathgen.stats.ox.ac.uk/impute/data_download_1000G_phase1_integrated.html). For association testing on the imputed variants, we performed missing data likelihood score tests with the SNPTEST v2 package [29]. An estimation of phenotypic variance explained by independent SNPs was performed with GCTA software [31]. We tested whether there is any interaction between independent SNPs in genes *KCNMA1* and *KCNQ5* via epistasis test implemented in PLINK [26].

## Results

There were 230 CRS cases and 1,594 controls in the African American cohort and 598 cases and 3,489 controls in the Caucasian group. Mean ages were similar between groups. Overall, 69% of African American and 60% of Caucasian CRS cases were diagnosed with asthma; 48% of the cases in the African American cohort and 62% of the cases in the Caucasian cohort had atopic syndrome. CRS cases were more likely to have a clinical diagnosis of otitis media. Both CRS groups were also more likely than controls to have undergone placement of tympanostomy tubes or surgical intervention for CRS-either functional endoscopic sinus surgery or adenoidectomy (Table 1). P-values<0.005 in both Caucasian and African American cohorts for the above comparisons between cases and controls.

**Table 1:** Demographics and clinical features of study groups.

	African American Cohort		Caucasian Cohort	
	Cases	Controls	Cases	Controls
Age (years)	11.7±5.2	9.5±5.8	12.1±4.7	10.8±5.8
Otitis Media (%)	48.26	21.59	50.92	9.81
Ear Tube (%)	15.22	0.63	25.96	1.49
Sinus Surgery (%)	29.57	4.39	42.21	6.0
FESS (%)	1.30	0	7.20	0.20
Adenoidectomy (%)	28.70	4.39	39.70	5.82

For Age, mean ± standard deviation is shown.

The complete list of analyzed genes encoding airway potassium channels is shown in Table 2. To determine if any genes were associated with the diagnosis of CRS we performed a PLINK set-based analysis as described above. In the Caucasian cohort, the locus yielding the strongest signal was at the *KCNMA1* gene, with an empirical p-value = 0.022 after multiple testing correction (Table 3).

**Table 2:** List of analyzed potassium channels.

Name	Gene	Locus	Reference
***Six-TMD, voltage-dependent K+ channels***			
Kv1.1	KCNA1	12p13.32	[44]
Kv1.3	KCNA3	1p13.3	[44]
Kv1.4	KCNA4	11p14	[44]
Kv1.5	KCNA5	12p13	[45]
Kv1.7	KCNA7	19q13.3	[45]
Kv2.2	KCNB2	8q	[44]
Kv4.1	KCND1	Xp11.23	[44]
Kv4.2	KCND2	7q31	[44]
Kv4.3	KCND3	1p13.3	[44]
Kv6.1	KCNG1	20q13	[45]
KvLQT1 (Kv7.1)	KCNQ1	11p15.5	[46], [47]
Kv7.2	KCNQ2	20q13.3	[48]
Kv7.3	KCNQ3	8q24	[48]
Kv7.4	KCNQ4	1p34	[48]
Kv7.5	KCNQ5	6q14	[48]
Kv9.3	KCNS3	2p24	[44]
***β-subunits***			
MiRP1	KCNE2	21q22.12	[49]
MiRP2	KCNE3	11q13-q14	[49]
MiRP3	KCNE4	2q36.3	[50]
KChIP2	KCNIP2	10q24	[44]
KChIP3	KCNIP3	2q21.1	[44]
Kvβ1	KCNAB1	3q26.1	[44]
Kvβ2	KCNAB2	1q36.3	[44]
Kvβ3	KCNAB3	17p13.1	[44]
***Six-TMD, Ca2+ activated K+ channels***			
SK1	KCNN1	19q13.1	[51]
KCa3.1	KCNN4	19q13.2	[46]
BKca	KCNMA1	10q22.3	[52], [53]
***Two-TMD, inward-rectified K^+^ channels***			
Kir2.1	KCNJ2	17q23-1q24.2	[54]
Kir3.1 GIRK1	KCNJ3	2q24.1	[55]
Kir3.2 GIRK2	KCNJ6	21q22.13-q22.2	[55]
Kir3.3 GIRK3	KCNJ9	1q21-q23	[55]
Kir3.4 GIRK4	KCNJ5	11q24	[55]
Kir4.2	KCNJ15	21q22.2	[56]
Kir6.1	KCNJ8	12p11.23	[56]
Kir7.1	KCNJ13	2q37	[57]
***Four-TMD, 2 pore K^+^ channels***			
Twik 1	KCNK1	1q42-q43	[58]
Twik 2	KCNK6	19q13.1	[58]
Trek 1	KCNK2	1q41	[58]
Trek 2	KCNK10	14q31	[58]
Task 2	KCNK5	6p21	[58]
Task 3	KCNK9	8q24.3	[58]
Task 4	KCNK17	6p21.2-p21.1	[58]
Thik 1	KCNK13	14q31-q32	[58]
KCNK7	KCNK7	11q13	[58]

**Table 3:** Results of gene set-based analyses of 44 potassium channel genes.

					Caucasian					African American				
Gene	Chr	Start (bp, hg18)	End (bp, hg18)	Size (kb)	NSNP	NSIG	ISIG	P-value	Adj. P-value	NSNP	NSIG	ISIG	P-value	Adj. P-value
KCNA1	12	4889333	4897683	8.35	12	0	0	1	1	11	0	0	1	1
KCNA3	1	111015832	111019178	3.35	6	0	0	1	1	6	0	0	1	1
KCNA4	11	29988340	29995064	6.72	10	0	0	1	1	10	0	0	1	1
KCNA5	12	5023345	5026210	2.87	33	0	0	1	1	31	0	0	1	1
KCNA7	19	54262486	54268010	5.52	9	0	0	1	1	7	0	0	1	1
KCNAB1	3	157321030	157739621	418.59	92	0	0	1	1	96	1	1	0.18	1
KCNAB2	1	6008966	6083110	74.14	14	0	0	1	1	14	0	0	1	1
KCNAB3	17	7766751	7773478	6.73	4	0	0	1	1	4	0	0	1	1
KCNB2	8	73612179	74013138	400.96	120	0	0	1	1	121	1	1	0.19	1
KCND1	X	48703582	48713195	9.61	3	0	0	1	1	3	0	0	1	1
KCND2	7	119700957	120177623	476.67	59	2	2	0.23	1	63	0	0	1	1
KCND3	1	112119976	112333300	213.32	112	1	1	0.20	1	115	1	1	0.56	1
KCNE2	21	34658192	34665310	7.12	14	0	0	1	1	13	0	0	1	1
KCNE3	11	73843533	73856248	12.72	16	0	0	1	1	17	0	0	1	1
KCNE4	2	223625105	223628599	3.49	7	0	0	1	1	9	0	0	1	1
KCNG1	20	49053599	49073082	19.48	8	0	0	1	1	8	0	0	1	1
KCNIP2	10	103575720	103593667	17.95	5	0	0	1	1	5	0	0	1	1
KCNIP3	2	95326798	95415552	88.75	8	0	0	1	1	8	0	0	1	1
KCNJ13	2	233339103	233349519	10.42	8	0	0	1	1	6	0	0	1	1
KCNJ15	21	38550533	38595616	45.08	17	0	0	1	1	19	0	0	1	1
KCNJ2	17	65677270	65687778	10.51	7	0	0	1	1	8	0	0	1	1
KCNJ3	2	155263338	155421260	157.92	47	0	0	1	1	49	0	0	1	1
KCNJ5	11	128266522	128293161	26.64	22	0	0	1	1	22	0	0	1	1
KCNJ6	21	37918656	38210566	291.91	107	0	0	1	1	109	0	0	1	1
KCNJ8	12	21809155	21819014	9.86	1	0	0	1	1	2	0	0	1	1
KCNJ9	1	158317983	158325836	7.85	10	0	0	1	1	10	0	0	1	1
KCNK1	1	231816372	231874607	58.24	42	0	0	1	1	43	0	0	1	1
KCNK10	14	87720998	87863004	142.01	51	0	0	1	1	53	0	0	1	1
KCNK13	14	89597860	89721948	124.09	33	0	0	1	1	34	0	0	1	1
KCNK17	6	39374754	39390214	15.46	23	0	0	1	1	22	0	0	1	1
KCNK2	1	213245507	213477059	231.55	43	0	0	1	1	47	1	1	0.26	1
KCNK5	6	39264724	39305229	40.51	22	1	1	0.027	1	20	3	2	0.027	1
KCNK6	19	43502323	43511489	9.17	6	0	0	1	1	6	0	0	1	1
KCNK7	11	65116901	65120043	3.14	3	0	0	1	1	3	0	0	1	1
KCNK9	8	140693985	140784481	90.5	45	0	0	1	1	44	2	2	0.042	1
KCNMA1	10	78299367	79067583	768.22	224	2	2	**0.0005**	**0.022**	227	1	1	0.43	1
KCNN1	19	17923110	17970930	47.82	18	0	0	1	1	18	0	0	1	1
KCNN4	19	48962524	48977249	14.73	11	0	0	1	1	11	0	0	1	1
KCNQ1	11	2422796	2826916	404.12	123	2	2	0.14	1	124	1	1	0.49	1
KCNQ2	20	61507985	61574437	66.45	14	0	0	1	1	14	0	0	1	1
KCNQ3	8	133210437	133562186	351.75	140	1	1	0.44	1	140	2	2	0.5	1
KCNQ4	1	41022270	41076947	54.68	23	0	0	1	1	23	0	0	1	1
KCNQ5	6	73388555	73962301	573.75	135	2	2	0.38	1	145	2	2	**0.0016**	**0.0704**
KCNS3	2	17923425	17977706	54.28	22	0	0	1	1	21	0	0	1	1

NSNP: number of SNPs within the gene and its upstream and downstream 20 kb genomic region; NSIG: number of significant SNPs; ISIG: number of independent significant SNPs; adj. P-value: P-value adjusted for multiple testing.

In the smaller African American cohort, *KCNQ5* demonstrated the strongest association, although it did not reach strict statistical significance (nominal p-value = 0.0016, corrected p-value = 0.0704 after multiple-testing correction).

In addition, two other genes were also associated with CRS with nominal p-value<0.05 (p = 0.027 for *KCNK5* in both Caucasian cohort and African American cohort; p = 0.042 for *KCNK9* in the African American cohort).

Detailed information on the independent significant SNPs for *KCNMA1* and *KCNQ5* are shown in Table 4. They are all located within the intron of their respective genes. Interestingly, the minor allele A of rs7900261 in *KCNMA1* renders a protective effect for the development of CRS (OR = 0.82). It is estimated that 0.9% of the phenotypic variance in CRS occurrence is explained by the two independent significant SNPs in *KCNMA1*. Similarly, the two independent significant SNPs in *KCNQ5* explained about 1% of the variance in CRS occurrence. We further investigated but found no interaction between independent significant SNPs in genes *KCNMA1* and *KCNQ5* respectively (Table S2). Allelic test results for all SNPs in these two genes are presented in Table S3.

**Table 4:** Significantly associated SNPs in genes *KCNMA1* and *KCNQ5*.

Cohort	Gene	SNP	Chr	bp(hg18)	Function	Minor/Major Allele	MAF (cases)	MAF (controls)	OR	SE	P-value
**Caucasian**	KCNMA1	rs2917454	10	78562421	intron	G/A	0.09	0.05	1.84	0.12	1.39×10^−7^
		rs7900261	10	78419202	intron	A/G	0.36	0.41	0.82	0.06	2.91×10^−3^
**African American**	KCNQ5	rs6907229	6	73940870	intron	C/T	0.28	0.18	1.74	0.11	9.32×10^−7^
		rs9343015	6	73943714	intron	C/T	0.56	0.47	1.41	0.10	6.29×10^−4^

SNP = single nucleotide polymorphism; Chr = chromosome; bp = base pair; MAF = minor allele frequency; OR = odds ratio; SE = standard error.

We also performed imputation analysis and identified an additional 19 variants in *KCNMA1* in LD with SNP rs2917454 associated with CRS (p-value<10^−4^) in the Caucasian cohort (Table 5). Further, we found 9 imputed variants in LD with rs6907229 in *KCNQ5* were also associated with CRS (p-value<10^−4^) in the African American cohort (Table 6).

**Table 5:** Imputed variants in gene *KCNMA1* with p-value<10^−4^ in the Caucasian cohort.

Variant	Chr	Pos (hg19)	Effect Allele	Reference Allele	Effect Allele Frequency	OR	95% CI	P-value
rs2250841	10	78910042	T	G	0.052	1.85	1.47, 2.34	1.85×10^−7^
rs1871063	10	78899404	T	C	0.053	1.84	1.46, 2.31	2.05×10^−7^
rs2766619	10	78897627	T	C	0.054	1.83	1.45, 2.31	2.36×10^−7^
rs2766624	10	78893799	C	T	0.054	1.83	1.45, 2.31	2.41×10^−7^
rs2616645	10	78889487	G	A	0.034	1.94	1.47, 2.57	1.41×10^−6^
rs1871064	10	78899595	A	G	0.029	1.99	1.47, 2.69	4.09×10^−6^
chr10:78889552:I	10	78889552	GA	G	0.033	1.91	1.44, 2.55	5.41×10^−6^
rs2574805	10	78905128	A	G	0.029	1.96	1.45, 2.65	6.49×10^−6^
rs2616650	10	78901708	A	G	0.029	1.95	1.44, 2.64	7.10×10^−6^
chr10:78894143:I	10	78894143	AC	A	0.032	1.90	1.42, 2.53	7.81×10^−6^
rs2766620	10	78899807	A	T	0.029	1.94	1.44, 2.63	7.88×10^−6^
chr10:78894142:I	10	78894142	CAA	C	0.033	1.89	1.42, 2.52	9.02×10^−6^
rs2574799	10	78894351	T	A	0.033	1.89	1.42, 2.52	9.20×10^−6^
rs2574797	10	78897797	T	A	0.030	1.92	1.43, 2.60	1.05×10^−5^
rs2616647	10	78894478	T	G	0.030	1.92	1.42, 2.60	1.08×10^−5^
rs2925826	10	78892469	G	A	0.030	1.92	1.42, 2.60	1.10×10^−5^
chr10:78894141:I	10	78894141	TCA	T	0.033	1.87	1.40, 2.49	1.31×10^−5^
rs11002022	10	78878445	T	C	0.023	1.96	1.40, 2.75	4.79×10^−5^
rs11002021	10	78877806	A	G	0.024	1.90	1.36, 2.65	8.45×10^−5^

Chr = chromosome; Pos = Position; OR = odds ratio; CI = confidence interval.

**Table 6:** Imputed variants in gene *KCNQ5* with p-value<10^−4^ in the African American cohort.

Variant	Chr	Pos (hg19)	Effect Allele	Reference Allele	Effect Allele Frequency	OR	95% CI	P-value
rs9351980	6	73885135	A	T	0.806	0.57	0.46, 0.71	8.14×10^−7^
chr6:73879813:D	6	73879813	A	AG	0.033	2.50	1.64, 3.81	9.32×10^−7^
rs1970549	6	73883761	A	G	0.804	0.57	0.46, 0.72	9.96×10^−7^
rs9351979	6	73880210	C	T	0.781	0.59	0.48, 0.74	1.89×10^−6^
rs2027545	6	73879839	A	G	0.781	0.60	0.48, 0.74	2.12×10^−6^
rs1970547	6	73881323	T	G	0.799	0.59	0.47, 0.74	2.81×10^−6^
rs2350386	6	73887606	C	G	0.878	0.58	0.45, 0.75	2.91×10^−5^
rs7756501	6	73886305	A	G	0.643	0.67	0.55, 0.82	8.30×10^−5^
rs2350385	6	73891177	C	T	0.646	0.68	0.55, 0.82	8.87×10^−5^

Chr = chromosome; Pos = Position; OR = odds ratio; CI = confidence interval.

**Figure 1:**
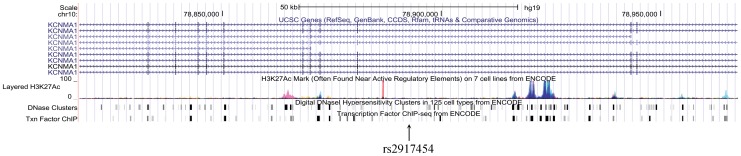
H3K27ac marks representing active regulatory elements found near the locus of rs2917454 in gene *KCNMA1*.

The two independent SNPs in *KCNMA1*, as well as those in *KCNQ5* are located in introns, therefore the polymorphisms of these variants do not have a direct effect on protein coding. However, intronic sequences may have an impact on several aspects of gene transcription [32]. Recently released results from the ENCODE project revealed a huge number of functional elements in the human genome [33], including many residing within introns. The NIH Roadmap Epigenomics project has also provided an enormous resource of epigenomic data [34]. By assessing the significant loci of these genes using the Integrated Regulation from ENCODE track in the UCSC genome browser, we found clear H3K27ac modification marks in close proximity to rs2917454 in *KCNMA1*(Figure 1). We also performed genomic annotation with the ENCODE database and Roadmap Epigenomics data via HaploReg software [35]. The results for the Caucasian and African American cohorts are presented in Tables 7 and 8, respectively. Interestingly, the results demonstrated that SNP rs2917454 overlaps with a glucocorticoid receptor binding motif. The other identified SNP of *KCNMA1* (rs7900261) overlaps with an enhancer histone mark and EWSR1-FLI1 motif suggesting a possible role in the regulation of gene expression. Additional neighboring SNPs, which are in perfect LD with rs2917454 and rs7900261, also overlap with enhancer histone marks, P300 binding sites and other transcription factor motifs (Table 7). Examination of SNPs in *KCNQ5* reveals similar associations with multiple promoter and enhancer histone marks as well as transcription factor binding motifs which have overlap with neighboring SNPs in good LD with rs6907229 and rs9343015 (Table 8).

**Table 7:** Regulatory elements at the significant loci in gene *KCNMA1*.

SNP	Chr	Pos(hg19)	r^2^	D′	Enhancer histone marks	DNAse hypersensitivity sites	Binding Proteins	Regulatory Motifs
**rs2917454**	10	78892415	1	1	.			GR
rs2766624	10	78893799	1	1	1 cell type			Pax-8
rs2766619	10	78897627	1	1	6 cell types			Nanog
rs1871063	10	78899404	1	1	3 cell types	AG04449,AoAF		MAZ
rs2250841	10	78910042	0.97	1	11 cel types		P300	EWSR1-FLI1;Pax-4;SP2
**rs7900261**	10	78749196	1	1	1 cell type	HGF		EWSR1-FLI1
rs3781153	10	78750074	1	1	1 cell type			HMG-IY;Hsf;STAT

r^2^ and D′ are measures of LD between the indicated SNP and the significant SNPs rs2917454 or rs7900261.

**Table 8:** Regulatory elements at the significant loci in gene *KCNQ5*.

SNP	Chr	Pos(hg19)	r^2^	D′	Promoter histone marks	Enhancer histone marks	DNAse hypersensitivity sites	Regulatory Motifs
rs200877792	6	73873577	0.82	0.95			Hepatocytes	Foxa;HDAC2;Homez
rs947747	6	73875824	0.8	0.93				Irf;Pbx3;p300
rs2350387	6	73877424	0.8	0.93		1 cell type		Evi-1;Hoxb13;Irf;Pbx-1;SP1
rs7763514	6	73877796	0.8	0.93		2 cell types		LRH1
rs2027545	6	73879839	0.81	1	1 cell type	4 cell types		Evi-1;Foxp1
rs9351979	6	73880210	0.81	1		1 cell type		Pou3f2;Sox
rs1970547	6	73881323	0.9	1				CDP;HNF1;PLZF
rs1970549	6	73883761	0.85	0.95				COMP1;Foxj2;Foxk1;Sox
rs9351980	6	73885135	1	1				Ik-1;Ik-2;NRSF;Sin3Ak-20
**rs9343015**	6	73886993	1	1	1 cell type	3 cell types	GM06990	Pou2f2
rs2882405	6	73887759	1	1		2 cell types		Mef2
rs3068379	6	73887987	0.97	1		2 cell types		Foxp1;Hoxb9;TATA
rs2882404	6	73888146	1	1		1 cell type		Foxi1;Foxj2;Foxl1;Hoxa9;Nkx6-1;Pou2f2
rs3799280	6	73888451	1	1		1 cell type		.
rs6937253	6	73888970	1	1		.		E2A;Ets;Lmo2-complex;MZF1::1–4;RXRA;SIX5;TCF12;ZEB1;Znf143
rs71696488	6	73889424	1	1		2 cell types		Ncx

r^2^ and D′ are measures of LD between the indicated SNP and significant SNPs rs6907229 or rs9343015.

## Discussion

With this study we aimed to identify whether variation harbored within genes encoding potassium channels found in airway epithelia is associated with CRS. We identified two SNPS (rs2917454 and rs790026) at the *KCNMA1* locus that were associated with the development of CRS in a Caucasian pediatric population. Interestingly, in a smaller cohort of African American children, we also observed a suggestive association between variants in *KCNQ5* (rs6907229 and rs9343015) and CRS. Further analysis demonstrated additional SNPs in LD with them that were associated with CRS. The primary SNP in the Caucasian group was found to be associated with a glucocorticoid receptor binding site. Multiple other intronic SNPs were predicted to have an impact on chromatin remodeling or gene expression. These findings raise the possibility that airway potassium channels physiology may contribute to the pathogenesis of CRS.

The clinical spectrum of CRS is a broad and ill-defined clinical entity. Classification of patients with CRS is difficult due to our limited understanding of the disease process which does not allow us to classify patients based on specific cellular or molecular changes. In the case of CF, ion transport dysfunction within epithelial cells, in almost all cases leads to a severe phenotype of CRS, and is evidence of the importance of precise maintenance of electrochemical gradients at the apical epithelial membrane.

**Figure 2:**
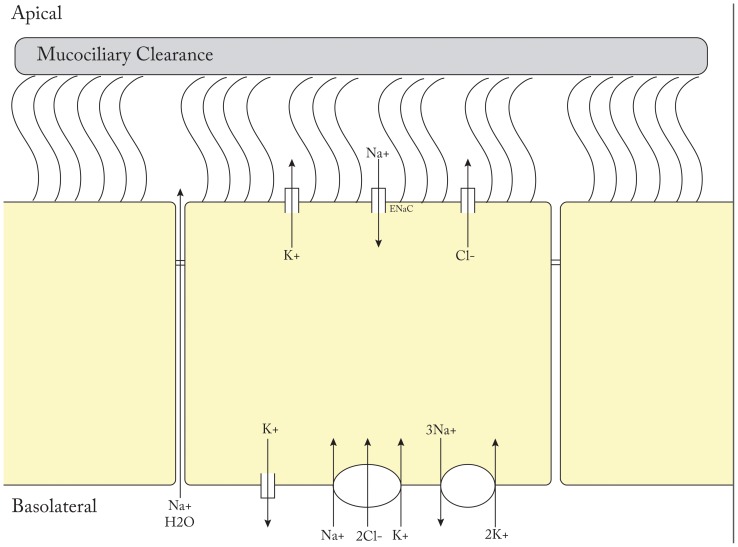
Schematic drawing of ciliated epithelial airway ion transport.

Mucociliary dysfunction is evident in essentially all patients with CRS and is a histological hallmark of the disease no matter the cause [36]. Both mucous hydration and cilia physiology are critical components of MCC [37]. Chloride anion transport is the primary ion current involved in the maintenance of air surface hydration, however, other channels contribute directly to or maintain gradients necessary for apical chloride secretion [38]. Fluid secretion models in secretory cells have supported the notion that chloride secretion is dependent on active transport mechanisms of other ions [15]. Intracellular accumulation of chloride anions is driven by the Na+K+2Cl− co-transporter present at the basolateral membrane of secretory cells where chloride ions can be concentrated up to 5-fold their normal electrochemical gradient. As a result, chloride ion movement can occur freely once chloride channels are activated at the apical membrane. Without electrical compensation for chloride efflux, this process would be inhibited, and basolateral potassium ion efflux into the interstitium plays an important role to support apical efflux of chloride ions. The apical sodium channel ENaC allows for trans-cellular transport of sodium ions and plays an important role in cell volume regulation as well as maintenance of periciliary height. Paracellular sodium movement through tight junctions balances the trans-epithelial gradient. In addition to the basolateral potassium channels discussed above, apical potassium channels have been identified and shown to contribute to chloride secretion and maintenance of ASL hydration [15]. A schematic drawing of epithelial ion transport is show in Figure 2. In the end, the regulation of ion and fluid transport at the apical surface of airway epithelium is a highly regulated system that continues to be elucidated.

The most convincing evidence that apical potassium channel physiology may directly affect MCC comes from a report by Manzanares et al. who found that BK channels were critical for the maintenance of adequate airway surface liquid volume, and inhibition of BK channels or knockdown of *KCNMA1* (BK alpha subunit) led to airway dehydration in NHBE cells and, most importantly, low ciliary beat frequency [22]. Other members of the apical potassium channel family have also been shown to play important roles in the regulation of ion transport as discussed above, but they have not been directly linked to regulation of air surface hydration, ciliary beat frequency, or MCC at this time. These findings together suggest that apical potassium channels within airway epithelial cells may play an important, previously underappreciated, role in supporting normal MCC. Further work needs to be done to understand their significance in the pathogenesis of CRS.

Although our study and previous work suggests the plausible role of apical potassium channel genes in CRS pathogenesis, only a small proportion of variance in CRS occurrence is explained by the independent significant SNPs in *KCNMA1* or *KCNQ5*. This is not surprising as CRS is a common, complex disease phenotype determined by multiple factors, including both genetic and environmental factors. A polygenic model is almost assured to be the underlying genetic mechanism.

Systemic or topical corticosteroids are an important treatment modality in patients with CRS. Corticosteroids are postulated to reduce mucosal eosinophil chemotaxis, increase eosinophil apoptosis, and prevent local histamine release. They decrease white blood cell migration, and reduce production of inflammatory mediators and antibodies [39]. Little is known about the role of glucocorticoids in ion transport. There is some evidence, however, that glucocorticoids can induce ENaC expression in lung epithelia [40]. The finding of an association between our significantly identified SNP in the Caucasian cohort with a glucocorticoid receptor site raises the possibility of a previously unrecognized therapeutic mechanism of these commonly used medications in the treatment of CRS. Further study on the role of steroid regulation on epithelial ion transport is required to test this genetic association.

Our additional identified SNPs in both cohorts were also located within introns and their biological impact may not be obvious, but they do demonstrate overlap with chromatin remodeling regions as well as gene expression enhancing and transcription factor binding sites. Intronic enhancers are not unusual. Chromatin looping and interaction between intronic enhancers and promoters have been identified for heme oxygenase-1 [41] and CFTR [42]. Additionally, alpha-fetoprotein has also been reported to harbor an enhancer and an alternative promoter in its first intron [43]. Therefore, many of the top SNPs found in our study or SNPs in good LD with them may possibly affect the risk of developing CRS through transcriptional regulation of *KCNMA1* or *KCNQ5*.

One of the limitations of this study is how the phenotype of CRS is defined. We selected a pediatric population in an effort to minimize environmental contributions to disease and enhance a genetic signal, however, the diagnosis of CRS in a pediatric population is more challenging than adults for multiple reasons including feasibility issues with nasal endoscopy and computed tomography (CT) imaging. Every effort was made to ensure patients met strict inclusion criteria for this study with not just a history suggestive of CRS, but objective data in the form of nasal endoscopy and/or CT imaging when feasible. Further, ruling out other known contributors of pediatric CRS including CF and primary immune deficiencies were sought to the best of our ability. Another major limitation is that these analyses are based on genetic associations. As always, functional studies should be performed to elucidate the biological consequences of these findings.

In summary, this is the first report of a genetic association between potassium channel epithelial physiology and the development of CRS. Further study is needed to confirm the biological relevance of this finding in both pediatric and adult CRS populations.

## Supporting Information

Table S1 **SNPs close to or in gene** ***CFTR*** **with p-value<1×10^−5^ before CF patients were removed and the corresponding statistics after CF patients were removed.**
(DOCX)

Table S2 **Test of interactions between independent significant SNPs in genes** ***KCNMA1*** **and** ***KCNQ5*** **.**
(DOCX)

Table S3 **Summary statistics of SNPs in the 44 potassium channel genes evaluated in this study.**
(XLSX)
