# Supplementary material for: Genetic Variation in Genes Encoding Airway Epithelial Potassium Channels Is Associated with Chronic Rhinosinusitis in a Pediatric Population
Source: PLoS One. 2014 Mar 3;9(3):e89329. doi: 10.1371/journal.pone.0089329 (PMC3940609; doi:10.1371/journal.pone.0089329)
Supplement: Table S1 — SNPs close to or in gene CFTR with p-value<1×10^−5^ before CF patients were removed and the corresponding statistics after CF patients were removed. (DOCX) [file pone.0089329.s001.docx]

Supplementary Table 1: SNPs close to or in gene *CFTR* with p-value < 1x10^-5^ before CF patients were removed and the corresponding statistics after CF patients were removed.

|  |  |  |  |  | include CF patients | | | exclude CF patients | | |
| --- | --- | --- | --- | --- | --- | --- | --- | --- | --- | --- |
| SNP | Closest Gene | Chr | bp(hg18) | Minor/ Major Allele | OR | SE | P-value | OR | SE | P-value |
| rs17544734 | CFTR | 7 | 116899358 | G/A | 1.70 | 0.0934 | 1.03x10^-8^ | 1.26 | 0.106 | 0.0305 |
| rs10487372 | CFTR | 7 | 116988135 | T/C | 1.57 | 0.0859 | 1.39x10^-7^ | 1.22 | 0.0954 | 0.0368 |
| rs10487368 | CFTR | 7 | 116927331 | T/C | 1.42 | 0.0720 | 8.28x10^-7^ | 1.22 | 0.0769 | 0.00908 |
| rs2237723 | CFTR | 7 | 116934783 | T/C | 1.42 | 0.0718 | 9.28x10^-7^ | 1.22 | 0.0767 | 0.00922 |
| rs2237724 | CFTR | 7 | 116941395 | A/G | 1.42 | 0.0720 | 9.71x10^-7^ | 1.22 | 0.0769 | 0.00989 |
| rs2027945 | CFTR | 7 | 116975058 | A/G | 1.42 | 0.0720 | 1.32x10^-6^ | 1.21 | 0.0769 | 0.0117 |
| rs213950 | CFTR | 7 | 116986769 | A/G | 1.31 | 0.0610 | 9.46x10^-6^ | 1.18 | 0.0633 | 0.00795 |
