# Supplementary material for: Genetic Variation in Genes Encoding Airway Epithelial Potassium Channels Is Associated with Chronic Rhinosinusitis in a Pediatric Population
Source: PLoS One. 2014 Mar 3;9(3):e89329. doi: 10.1371/journal.pone.0089329 (PMC3940609; doi:10.1371/journal.pone.0089329)
Supplement: Table S2 — Test of interactions between independent significant SNPs in genes KCNMA1 and KCNQ5 . (DOCX) [file pone.0089329.s002.docx]

Supplementary Table 2: Test of interactions between independent significant SNPs in genes *KCNMA1* and *KCNQ5.*

| Cohort | Gene | Chr | SNP1 | SNP2 | OR (interaction) | P-value |
| --- | --- | --- | --- | --- | --- | --- |
| Caucasian | KCNMA1 | 10 | rs7900261 | rs2917454 | 0.955 | 0.791 |
| African American | KCNQ5 | 6 | rs6907229 | rs9343015 | 1.25 | 0.280 |
